# Novel diagnostic processes and challenges in bronchoscopy

**DOI:** 10.3389/pore.2024.1611774

**Published:** 2024-05-21

**Authors:** Zsolt Pápai-Székely, Gábor Grmela, Veronika Sárosi

**Affiliations:** ^1^ Department of Pulmonology, Szent György University Teaching Hospital of Fejér County, Székesfehérvár, Hungary; ^2^ Department of Pulmonology, Pécs Medical School, Pécs, Hungary

**Keywords:** diagnostic bronchoscopy, respiratory medicine, endobronchial ultrasound, virtual bronchoscopy, autofluorescence bronchoscopy

## Abstract

Diagnostic bronchoscopy is a minimally invasive procedure that plays a crucial role in the diagnosis and management of various respiratory conditions. This paper explores the advancements in technology that have revolutionized the field and focuses on the new diagnostic procedures in bronchoscopy that have emerged in recent years. These innovative techniques have expanded the diagnostic capabilities of bronchoscopy, allowing for more accurate and comprehensive evaluation of respiratory conditions. This paper will also discuss the challenges in the diagnostic process with bronchoscope.

## Introduction

Diagnostic bronchoscopy is a minimally invasive procedure that allows for direct visualization and sampling of the airways, providing valuable diagnostic information in the evaluation of various respiratory conditions. Technological advancements have revolutionized the field of diagnostic bronchoscopy. Flexible bronchoscopes have replaced rigid instruments, enabling easier access to peripheral airways [1]. Endoscopic ultrasound techniques revolutionized the diagnostic work-up, due to their extreme importance we leave it to be discussed in a separate paper. Virtual bronchoscopy and navigation systems provide enhanced visualization and guidance during the procedure. Optical coherence tomography and confocal laser endomicroscopy offer real-time imaging of the airway mucosa, aiding in the detection of early neoplastic lesions. The field of diagnostic bronchoscopy continues to evolve, with ongoing research and development of advanced imaging techniques, biomarkers, and molecular testing. The integration of artificial intelligence in bronchoscopy holds promise for improving diagnostic accuracy and efficiency.

## Autofluorescence bronchoscopy (AFB)

Autofluorescence bronchoscopy (AFB) is a technique that utilizes the natural fluorescence properties of bronchial tissue to detect early changes associated with pre-malignant and malignant lesions. It involves the use of a specialized bronchoscope equipped with a light source that emits specific wavelengths of light to excite the fluorophores in the tissue.

### Indications and applications of AFB

AFB is primarily used for the detection and surveillance of pre-malignant and early-stage lung cancer. It can identify subtle changes in the bronchial mucosa that may not be visible under white light bronchoscopy. AFB is particularly useful in patients with a high risk of developing lung cancer, such as smokers or individuals with a history of occupational exposure to carcinogens [2].

In addition to lung cancer, AFB has shown promise in the evaluation of other respiratory conditions, such as bronchial dysplasia, chronic obstructive pulmonary disease (COPD), and interstitial lung diseases. It can aid in the early detection and characterization of these conditions, allowing for timely intervention and management.

### Technique and procedure of AFB

AFB is performed using a specialized bronchoscope that emits blue or ultraviolet light to excite the fluorophores in the bronchial tissue. The emitted fluorescence is then visualized and interpreted by a bronchoscopist. Areas of abnormal fluorescence, such as loss of autofluorescence or increased fluorescence intensity, may indicate the presence of pre-malignant or malignant lesions.

The procedure is typically performed under conscious sedation or general anesthesia, depending on the patient’s tolerance and the complexity of the case. AFB requires specialized training and expertise to accurately interpret the fluorescence patterns and differentiate between normal and abnormal findings.

## Confocal laser endomicroscopy (CLE)

Confocal laser endomicroscopy (CLE) is a real-time imaging technique that allows for microscopic visualization of the bronchial mucosa during bronchoscopy. It involves the use of a miniaturized confocal microscope probe that can be inserted through the working channel of a standard bronchoscope [3].

### Indications and applications of CLE

CLE is primarily used for the evaluation of bronchial mucosal abnormalities, such as pre-malignant lesions, inflammatory conditions, and infectious processes. It provides high-resolution images of the cellular and subcellular structures of the bronchial mucosa, allowing for detailed assessment and characterization of these abnormalities.

CLE has shown promise in the early detection and surveillance of lung cancer, as well as the evaluation of other respiratory conditions, such as asthma, COPD, and interstitial lung diseases. It can aid in the identification of specific cellular features, such as cellular atypia or inflammatory cell infiltrates, that may not be visible under white light bronchoscopy. It can be used as a guiding system to find optimal cryobiopsy location in interstitial lung diseases [4].

### Technique and procedure of CLE

CLE is performed using a specialized bronchoscope equipped with a confocal microscope probe. The probe is inserted through the working channel of the bronchoscope and positioned adjacent to the target area of interest. Laser light is then emitted from the probe and focused on the bronchial mucosa, while the emitted fluorescent signals are captured and processed to generate real-time microscopic images.

The procedure is typically performed under conscious sedation or general anesthesia, depending on the patient’s tolerance and the complexity of the case. CLE requires specialized training and expertise to accurately interpret the microscopic images and differentiate between normal and abnormal findings.

## Optical coherence tomography (OCT)

Optical coherence tomography (OCT) is an imaging technique that utilizes light waves to generate cross-sectional images of the bronchial mucosa. It provides high-resolution images of the tissue architecture, allowing for the assessment of cellular and structural abnormalities.

### Indications and applications of OCT

OCT is primarily used for the evaluation of bronchial mucosal abnormalities, such as pre-malignant lesions, inflammatory conditions, and airway remodeling. It can aid in the early detection and surveillance of lung cancer, as well as the evaluation of other respiratory conditions, such as asthma, COPD, and bronchiectasis.

OCT has also shown potential in guiding therapeutic interventions, such as laser ablation or photodynamic therapy, by providing real-time feedback on the depth and extent of tissue involvement. It can help optimize treatment planning and improve treatment outcomes.

### Technique and procedure of OCT

OCT is performed using a specialized bronchoscope equipped with an OCT imaging probe. The probe is inserted through the working channel of the bronchoscope and positioned adjacent to the target area of interest. Low-coherence light waves are emitted from the probe and directed onto the bronchial mucosa, while the reflected light is captured and processed to generate cross-sectional images.

The procedure is typically performed under conscious sedation or general anesthesia, depending on the patient’s tolerance and the complexity of the case. OCT requires specialized training and expertise to accurately interpret the cross-sectional images and differentiate between normal and abnormal findings.

New diagnostic procedures in bronchoscopy, such as autofluorescence bronchoscopy (AFB), confocal laser endomicroscopy (CLE), and optical coherence tomography (OCT), have expanded the diagnostic capabilities of bronchoscopy by providing real-time imaging and characterization of bronchial mucosal abnormalities. These techniques offer the potential for early detection, precise characterization, and targeted management of respiratory conditions, including pre-malignant and malignant lesions. Understanding the indications, techniques, and limitations of these new diagnostic procedures is essential for healthcare professionals involved in bronchoscopy and respiratory care.

## Ultrathin bronchoscopy: advancements in minimally invasive diagnostic techniques

Ultrathin bronchoscopy is a minimally invasive diagnostic technique that utilizes a thin and flexible bronchoscope to visualize and access the airways. This chapter explores the advancements in ultrathin bronchoscopy, its applications, benefits, and limitations in the field of respiratory medicine [5].

### Ultrathin bronchoscopy: an overview

Ultrathin bronchoscopy involves the use of a bronchoscope with a small diameter, typically ranging from 2.2 to 3.0 mm. This slim and flexible design allows for easier navigation through the airways, including the peripheral regions. The bronchoscopist can visualize the airways and perform diagnostic procedures with minimal discomfort to the patient.

### Applications of ultrathin bronchoscopy

#### Peripheral lung lesions

One of the primary applications of ultrathin bronchoscopy is the evaluation of peripheral lung lesions. These lesions are often challenging to access using traditional bronchoscopic techniques due to their location in the smaller airways. Ultrathin bronchoscopy provides improved maneuverability and visualization in these areas, allowing for targeted biopsies and sampling of peripheral lesions [6].

#### Airway assessment and management

Ultrathin bronchoscopy is also useful for assessing and managing various airway conditions. It can be used to evaluate airway stenosis, granulation tissue, and other abnormalities. Additionally, it allows for the placement of stents or other therapeutic interventions in the airways, providing relief for patients with obstructive airway diseases [7].

#### Pediatric bronchoscopy

Ultrathin bronchoscopy is particularly valuable in pediatric patients. The small diameter of the bronchoscope reduces discomfort and the risk of complications in children. It allows for thorough evaluation of the airways and facilitates diagnostic procedures, such as bronchoalveolar lavage and transbronchial lung biopsy, in pediatric populations.

### Benefits of ultrathin bronchoscopy

#### Minimally invasive procedure

Ultrathin bronchoscopy offers a minimally invasive alternative to traditional bronchoscopy. The small diameter of the bronchoscope reduces patient discomfort and the risk of complications, such as bleeding or pneumothorax. It allows for outpatient procedures and faster recovery times.

#### Improved access to peripheral lesions

The slim and flexible design of the ultrathin bronchoscope enables better access to peripheral lung lesions. It can navigate through narrow and tortuous airways, reaching areas that may be challenging to visualize and sample with larger bronchoscopes. This improves the diagnostic yield and reduces the need for more invasive procedures, such as surgical lung biopsy.

#### Enhanced patient tolerance

Ultrathin bronchoscopy is better tolerated by patients, especially those with sensitive airways or respiratory conditions. The small diameter and flexibility of the bronchoscope cause less irritation and discomfort during the procedure, making it more suitable for patients with compromised lung function or heightened airway sensitivity.

### Limitations and challenges of ultrathin bronchoscopy

#### Limited instrumentation and maneuverability

The small diameter of the ultrathin bronchoscope limits the availability of specialized instruments and accessories. This may restrict certain diagnostic and therapeutic procedures that require large instruments or tools. Additionally, the flexibility of the bronchoscope may limit its maneuverability in some cases.

#### Reduced visualization and image quality

Ultrathin bronchoscopes may have limitations in image quality and visualization compared to large bronchoscopes. The small diameter can result in reduced light transmission and image resolution. However, advancements in technology have led to improvements in image quality, mitigating this limitation to some extent.

#### Learning curve and expertise

Ultrathin bronchoscopy requires specialized training and expertise. The bronchoscopist must develop skills in navigating through the smaller airways and performing procedures with the limited instrumentation available. Adequate training and experience are crucial to ensure safe and effective use of ultrathin bronchoscopy.

#### Future directions and conclusion

Ultrathin bronchoscopy has emerged as a valuable tool in the field of respiratory medicine, offering a minimally invasive approach to diagnose and manage various airway conditions. Ongoing advancements in technology and instrumentation are expected to further improve the capabilities and image quality of ultrathin bronchoscopes. With continued research and training, ultrathin bronchoscopy has the potential to become a standard diagnostic technique, providing safer and more comfortable procedures for patients while maintaining diagnostic accuracy.

## Electromagnetic navigation bronchoscopy (ENB)

Electromagnetic navigation bronchoscopy (ENB) is a technique that allows for the navigation and sampling of peripheral lung lesions that are not easily accessible by conventional bronchoscopy. It utilizes electromagnetic technology to create a virtual 3D map of the patient’s airways, guiding the bronchoscope to the target lesion [8].

### Indications and applications of ENB

ENB is primarily used for the diagnosis and staging of peripheral lung lesions, particularly when other diagnostic modalities, such as CT-guided biopsy or surgical resection, are not feasible or desirable. It allows for the sampling of small or inaccessible lesions, providing valuable information for treatment planning and prognosis.

ENB can also be used for the placement of fiducial markers for radiation therapy, as well as the delivery of therapeutic agents, such as brachytherapy or photodynamic therapy, to peripheral lung lesions. It offers a less invasive alternative to surgical resection for selected patients with early-stage lung cancer.

### Technique and procedure of ENB

ENB involves the use of a specialized bronchoscope equipped with electromagnetic sensors and a working channel for biopsy instruments. Prior to the procedure, a CT scan of the patient’s chest is obtained, which is then used to create a virtual 3D map of the airways and target lesion.

During the procedure, the bronchoscope is navigated through the airways using real-time electromagnetic guidance. The virtual 3D map is overlaid onto the live bronchoscopic images, allowing the operator to accurately guide the bronchoscope to the target lesion. Biopsy instruments can then be advanced through the working channel to obtain tissue samples for diagnosis [9].

## Virtual bronchoscopy: a non-invasive approach to airway visualization and assessment

Virtual bronchoscopy is a non-invasive imaging technique that utilizes computed tomography (CT) or magnetic resonance imaging (MRI) data to create a three-dimensional virtual representation of the airways. This chapter explores the advancements in virtual bronchoscopy, its applications, benefits, and limitations in the field of respiratory medicine [10].

### Virtual bronchoscopy: an overview

Virtual bronchoscopy involves the use of advanced imaging software to generate a virtual model of the airways based on CT or MRI scans. The virtual model allows for a detailed visualization of the airway anatomy, providing valuable information for diagnostic and therapeutic purposes [11].

### Applications of virtual bronchoscopy

#### Airway assessment and pathology detection

Virtual bronchoscopy is primarily used for the assessment of airway anatomy and the detection of various pathologies. It allows for a comprehensive evaluation of the airways, including the detection of tumors, stenosis, strictures, and other abnormalities. Virtual bronchoscopy can aid in the diagnosis and planning of treatment for conditions such as lung cancer, bronchiectasis, and tracheobronchomalacia.

#### Preoperative planning and simulation

Virtual bronchoscopy is valuable in preoperative planning for airway interventions. It enables the bronchoscopist to assess the feasibility and optimal approach for procedures such as bronchial stenting, laser therapy, or endobronchial valve placement. Virtual bronchoscopy can simulate the procedure, allowing for precise planning and reducing the risk of complications.

#### Patient education and communication

Virtual bronchoscopy provides a visual representation of the airways that can be easily understood by patients. It can be used as a tool for patient education, allowing them to visualize their airway condition and understand the proposed treatment plan. Virtual bronchoscopy enhances communication between the healthcare provider and the patient, leading to better patient engagement and informed decision-making.

### Benefits of virtual bronchoscopy

#### Non-invasive and radiation-free

Virtual bronchoscopy is a non-invasive imaging technique that does not require the insertion of a bronchoscope into the airways. This eliminates the need for sedation or anesthesia and reduces the risk of complications associated with invasive procedures. Additionally, virtual bronchoscopy does not involve ionizing radiation, making it a safer alternative to traditional bronchoscopy or CT scans.

#### Comprehensive airway visualization

Virtual bronchoscopy provides a comprehensive visualization of the airways, allowing for a detailed assessment of the anatomy and pathology. The three-dimensional virtual model offers a panoramic view of the airways, enabling the bronchoscopist to explore different angles and perspectives. This enhances the diagnostic accuracy and aids in treatment planning.

#### Time and cost efficiency

Virtual bronchoscopy can be performed using existing CT or MRI scans, eliminating the need for additional imaging procedures. This saves time and reduces healthcare costs associated with multiple imaging studies. Virtual bronchoscopy also allows for efficient preoperative planning, optimizing the use of resources and minimizing procedural delays.

### Limitations and challenges of virtual bronchoscopy

#### Limited functional information

Virtual bronchoscopy provides detailed anatomical information but lacks functional data. It cannot assess dynamic airway collapse, airflow obstruction, or mucosal abnormalities that may be observed during traditional bronchoscopy. Therefore, virtual bronchoscopy should be used in conjunction with other diagnostic modalities to obtain a comprehensive evaluation of the airways.

#### Dependence on high-quality imaging

The accuracy and reliability of virtual bronchoscopy depend on the quality of the CT or MRI scans used to generate the virtual model. Suboptimal image quality, artifacts, or motion artifacts can affect the accuracy of the virtual model and limit its diagnostic value. Therefore, it is essential to ensure high-quality imaging for optimal results.

#### Operator experience and interpretation

Virtual bronchoscopy requires expertise in image interpretation and manipulation. The bronchoscopist must be familiar with the software and techniques used to generate and navigate the virtual model. Adequate training and experience are necessary to accurately interpret the virtual bronchoscopy images and make informed clinical decisions.

#### Future directions and conclusion

Virtual bronchoscopy has emerged as a valuable non-invasive tool in the field of respiratory medicine, providing detailed visualization of the airways and aiding in diagnosis, treatment planning, and patient communication. Ongoing advancements in imaging technology and software algorithms are expected to further enhance the capabilities of virtual bronchoscopy. With continued research and development, virtual bronchoscopy has the potential to become an integral part of the diagnostic and therapeutic armamentarium, improving patient care and outcomes in respiratory medicine.

## Cone beam CT in bronchoscopy: advancements in imaging and navigation

Cone Beam CT (CBCT) is a three-dimensional imaging technique that has revolutionized the field of bronchoscopy. This chapter explores the advancements in CBCT technology, its applications, benefits, and limitations in the context of bronchoscopy [12].

### Cone beam CT: an overview

Cone Beam CT is a specialized imaging technique that utilizes a cone-shaped X-ray beam and a flat-panel detector to capture high-resolution, three-dimensional images of the airways. Unlike traditional CT scans, CBCT provides real-time imaging during bronchoscopy procedures, allowing for improved navigation and accurate localization of lesions.

### Applications of cone beam CT in bronchoscopy

#### Localization of peripheral lung lesions

One of the primary applications of CBCT in bronchoscopy is the localization of peripheral lung lesions. CBCT provides real-time imaging of the airways and allows the bronchoscopist to precisely locate and target lesions that may be difficult to visualize with traditional bronchoscopic techniques. This improves the accuracy of diagnostic procedures and reduces the need for additional invasive interventions.

#### Guidance for biopsy and sampling

CBCT can guide the bronchoscopist during biopsy and sampling procedures. The real-time imaging provided by CBCT helps in accurately positioning the bronchoscope and instruments, ensuring optimal sampling of the target lesion. This improves the diagnostic yield and reduces the risk of complications associated with blind biopsies.

#### Assessment of airway anatomy and pathology

CBCT allows for detailed assessment of airway anatomy and pathology. It provides high-resolution images that can reveal structural abnormalities, such as stenosis, strictures, or tumors, which may not be easily visible with traditional bronchoscopic techniques. This aids in pre-procedural planning and enhances the bronchoscopist’s understanding of the patient’s airway condition.

### Benefits of cone beam CT in bronchoscopy

#### Real-time imaging and navigation

The real-time imaging capability of CBCT provides immediate feedback to the bronchoscopist during the procedure. This allows for precise navigation through the airways, reducing the risk of complications and improving the efficiency of the procedure. The bronchoscopist can visualize the position of the bronchoscope and instruments in relation to the target lesion, ensuring accurate sampling and minimizing damage to healthy tissue.

#### Improved lesion localization

CBCT enables accurate localization of peripheral lung lesions, even in challenging anatomical locations. The three-dimensional imaging provides a clear visualization of the lesion’s position relative to the airway, facilitating targeted biopsies and reducing the need for additional procedures.

#### Reduced radiation exposure

Compared to traditional CT scans, CBCT in bronchoscopy typically involves lower radiation doses. The cone-shaped X-ray beam used in CBCT focuses on the area of interest, minimizing radiation exposure to surrounding tissues. This is particularly beneficial for patients who require multiple imaging procedures or have a higher risk of radiation-related complications.

### Limitations and challenges of cone beam CT in bronchoscopy

#### Equipment and infrastructure requirements

CBCT requires specialized equipment and infrastructure, including a dedicated CBCT system and a flat-panel detector. These requirements may limit the availability of CBCT in certain healthcare settings, particularly smaller clinics or facilities with limited resources.

#### Learning curve and interpretation

The interpretation of CBCT images requires specialized training and expertise. Bronchoscopists need to develop skills in navigating and interpreting three-dimensional images to effectively utilize CBCT during procedures. Adequate training and experience are essential to ensure accurate interpretation and optimal utilization of CBCT in bronchoscopy.

#### Cost considerations

The cost of CBCT equipment and maintenance can be a limiting factor for widespread adoption. The initial investment and ongoing expenses associated with CBCT may pose challenges for healthcare institutions, particularly those with limited budgets.

#### Future directions and conclusion

Cone Beam CT has emerged as a valuable tool in bronchoscopy, providing real-time imaging and navigation capabilities. Ongoing advancements in CBCT technology, such as improved image quality and reduced radiation doses, are expected to further enhance its utility in the field. With continued research and development, CBCT has the potential to become a standard imaging modality in bronchoscopy, enabling more accurate diagnoses, targeted interventions, and improved patient outcomes.

## Robotic bronchoscopy: advancements in diagnostic techniques

Robotic bronchoscopy is an emerging technology that combines the precision of robotics with the diagnostic capabilities of bronchoscopy. This chapter explores the advancements in robotic bronchoscopy, its applications, benefits, and limitations in the field of respiratory medicine. Robotic bronchoscopy involves the use of a robotic system to navigate and manipulate a bronchoscope within the airways. The robotic system consists of a robotic arm, a control console, and specialized instruments. The bronchoscopist operates the system from the console, which provides a 3D visualization of the airways and precise control of the robotic arm [13].

### Applications of robotic bronchoscopy

#### Peripheral lung lesions

One of the primary applications of robotic bronchoscopy is the diagnosis and management of peripheral lung lesions. These lesions are often challenging to access using traditional bronchoscopic techniques. Robotic bronchoscopy allows for improved navigation and maneuverability within the peripheral airways, enabling the bronchoscopist to reach and sample these lesions more effectively [14, 15].

#### Mediastinal lymph node sampling

Robotic bronchoscopy also offers advantages in mediastinal lymph node sampling. The robotic system provides enhanced visualization and precise control, allowing for accurate targeting and sampling of lymph nodes. This is particularly beneficial in cases where lymph nodes are in difficult-to-reach areas or when there is a need for precise sampling for staging of lung cancer.

#### Interventional procedures

In addition to diagnostic purposes, robotic bronchoscopy can be used for interventional procedures. The robotic system enables the bronchoscopist to perform procedures such as endobronchial biopsy, endobronchial ultrasound-guided transbronchial needle aspiration (EBUS-TBNA), and laser ablation with increased precision and control. This opens new possibilities for minimally invasive treatment options [16].

### Benefits of robotic bronchoscopy

#### Improved access and navigation

Robotic bronchoscopy provides improved access to peripheral lung lesions and challenging anatomical locations. The robotic arm can navigate through narrow and tortuous airways with greater precision, reducing the risk of complications and improving the diagnostic yield.

#### Enhanced visualization

The 3D visualization provided by the robotic system enhances the bronchoscopist’s ability to visualize the airways and target lesions. This improved visualization allows for more accurate targeting and sampling, leading to better diagnostic outcomes.

#### Increased precision and control

The robotic system offers increased precision and control during bronchoscopy procedures. The robotic arm can perform precise movements and manipulations, reducing the risk of tissue damage and improving the safety of the procedure.

### Limitations and challenges of robotic bronchoscopy

#### Cost and availability

One of the main limitations of robotic bronchoscopy is its cost and availability. The robotic systems and associated instruments are expensive, making them less accessible in certain healthcare settings. Additionally, the expertise required to operate the robotic system may be limited to specialized centers with trained personnel.

#### Learning curve

Robotic bronchoscopy requires specialized training and expertise. The learning curve for mastering the robotic system and its associated techniques can be steep. Adequate training and proctoring are essential to ensure safe and effective use of the technology.

#### Technical limitations

While robotic bronchoscopy offers many advantages, it also has some technical limitations. The size of the robotic arm and instruments may limit access to certain areas of the airways. Additionally, the robotic system may not be suitable for all patients, such as those with severe airway abnormalities or significant comorbidities.

#### Future directions and conclusion

Robotic bronchoscopy is a promising advancement in the field of respiratory medicine. As technology continues to evolve, we can expect further improvements in robotic systems, including miniaturization of instruments and enhanced capabilities. With ongoing research and development, robotic bronchoscopy has the potential to revolutionize the diagnostic and interventional management of respiratory conditions, providing safer and more precise procedures for patients.

## Challenges in the diagnostic process of bronchoscopy

Bronchoscopy is a valuable diagnostic tool for evaluating respiratory conditions. However, the diagnostic process of bronchoscopy is not without challenges. We will discuss some of the common challenges encountered during bronchoscopy and strategies to overcome them, ensuring a successful diagnostic outcome.

### Technical challenges

#### Limited access to peripheral lesions

One of the major challenges in bronchoscopy is accessing peripheral lung lesions. These lesions are located deep within the lung tissue and may be difficult to reach using a standard bronchoscope. Traditional bronchoscopic techniques, such as transbronchial biopsy, may have limited success in obtaining adequate tissue samples from these lesions.

To overcome this challenge, various advanced techniques have been developed, including electromagnetic navigation bronchoscopy (ENB) and radial endobronchial ultrasound (EBUS). These techniques provide real-time imaging guidance, allowing for accurate navigation to peripheral lesions and increasing the diagnostic yield.

#### Inadequate visualization

Another challenge in bronchoscopy is inadequate visualization of the airway and target lesions. Factors such as excessive secretions, blood, or poor lighting can hinder the bronchoscopist’s ability to clearly visualize the airway and obtain accurate diagnostic information.

To address this challenge, proper airway preparation is essential. Pre-bronchoscopy measures, such as bronchial hygiene techniques and administration of mucolytic agents, can help reduce secretions and improve visualization. Adequate suctioning and irrigation during the procedure can also help clear the airway and improve visualization.

### Diagnostic challenges

#### Sampling error

Sampling error is a common challenge in bronchoscopy, particularly when obtaining tissue samples for histopathological examination. The bronchoscopist must ensure that the biopsy samples are representative of the target lesion to obtain an accurate diagnosis.

To minimize sampling error, it is important to carefully select the biopsy site based on radiological findings and bronchoscopic assessment. Techniques such as transbronchial needle aspiration (TBNA) and endobronchial ultrasound-guided transbronchial needle aspiration (EBUS-TBNA) can provide more targeted sampling of mediastinal lymph nodes and peripheral lesions, improving the diagnostic yield.

#### False-negative results

Obtaining false-negative results is another challenge in bronchoscopy. In some cases, the bronchoscopist may not be able to visualize or sample the lesion adequately, leading to a negative diagnostic outcome despite the presence of pathology.

To address this challenge, a multidisciplinary approach is crucial. Collaboration with radiologists, pathologists, and other specialists can help correlate clinical, radiological, and pathological findings to ensure a comprehensive diagnostic evaluation. Repeat bronchoscopy or alternative diagnostic procedures may be considered if there is a high suspicion of pathology despite initial negative results.

### Safety challenges

#### Complications and adverse events

Bronchoscopy, like any invasive procedure, carries the risk of complications and adverse events. These can range from minor complications such as bleeding or pneumothorax to more serious events such as respiratory distress or cardiac arrhythmias.

To mitigate these risks, it is important to adhere to strict safety protocols and guidelines. Proper patient selection, thorough pre-procedure assessment, and appropriate monitoring during and after the procedure are essential. Adequate training and expertise in bronchoscopy, as well as prompt recognition and management of complications, are crucial for ensuring patient safety.

#### Infection control

Infection control is a significant challenge in bronchoscopy due to the potential for cross-contamination and transmission of infectious agents. The bronchoscope and associated accessories can harbor bacteria or other pathogens, posing a risk of infection to both patients and healthcare providers.

To address this challenge, strict adherence to infection control practices is essential. This includes proper cleaning and disinfection or sterilization of bronchoscopes and accessories, adherence to hand hygiene protocols, and use of personal protective equipment. Regular monitoring and auditing of infection control practices can help identify and address any gaps or deficiencies.

## Conclusion

The diagnostic process of bronchoscopy is not without challenges. Technical challenges, such as limited access to peripheral lesions and inadequate visualization, can impact the diagnostic yield. Diagnostic challenges, including sampling error and false-negative results, require a multidisciplinary approach and careful consideration of alternative diagnostic procedures. Safety challenges, such as complications and infection control, necessitate adherence to strict protocols and guidelines. By recognizing and addressing these challenges, healthcare professionals can optimize the diagnostic process of bronchoscopy and improve patient outcomes.
